# *The Organon* and Materia Medica Club of the Bay Cities of California

**Published:** 1896-01

**Authors:** Eleanor F. Martin


### *THE ORGANON* AND MATERIA MEDICA CLUB OF THE BAY CITIES OF CALIFORNIA.

The regular semi-monthly meeting was held at the office of Dr. George H. Martin, 921 Polk Street, San Francisco, August 2d, 1895.

Members present were: Drs. J. M. Selfridge, George H. Martin, A. McNeil, S. E. Chapman, M. T. Wilson, J. E. Lilienthal, and C. M. Selfridge. Visitor, Dr. M. F. Underwood.

The meeting was called to order at 8.15 P. M. by the President, Dr. J. M. Selfridge. The minutes of the previous meeting were read, and after a few slight corrections by Drs. J. M. Selfridge and Wilson, were approved.

The President then appointed Dr. McNeil reader of the evening, who read from *The Organon*, Section 236.

### Discussion.

Dr. McNeil—This mode of administering the medicine is the best. The practice of giving the medicine just before or during the attack is worn out.

Sections 237 and 238 were then read.

### Discussion.

Dr. McNeil—These remarks are entirely too modest for Hahnemann. It is not necessary for the patient to leave the locality if the indicated remedy is given.

Dr. Chapman—I want to ask Dr. NcNeil if he has ever noticed this fact. I lived in Placer County, and while there I noticed that people would come up from the marshes who never had malaria, or only had it slightly, and yet would almost shake to death when they came to our place. The paroxysms would seem to increase as they left the malarial district.

Dr. McNeil—That is very plain. A person living in a thoroughly malarious region takes Quinine, and the vital forces are so lowered that he cannot form a paroxysm. There is a battle against the disease. Take a case in Marysville, and the vital powers cannot produce a clear case of intermittent fever. Send him to Placerville, where the air is pure, the vital forces will be restored, he recuperates, and the paroxysm returns. In a case where Quinine has been used to excess there will be the Quinine cachexia. In dumb ague, caused by malaria and Quinine, the patient is unable to have a clear paroxysm, but you give the indicated remedy and it will bring on a true paroxysm, and the patient will blame you for it.

Dr. J. M. Selfridge—A patient from Stockton came to Oakland. She had never had a chill. She was sallow and looked as though she had malaria. She married a minister. When she was six weeks in Oakland she commenced to have chills. As a rule chills do not originate in Oakland.

Dr. Chapman—The Doctor's explanation seems reasonable. I never could understand why these explosions occurred.

Dr. Martin—There is another explanation for this. A person living in a malarial district has had Quinine and malarial poisoning, and the system becomes accustomed to it. When they go away they are more likely to have the effects of the poison manifest themselves. When a person has been accustomed to drinking coffee they do not feel the bad effects of it, but when they leave it off they have the effect of coffee poisoning manifested by headaches, etc. Persons working in sewers live for years and are not affected by it, but when they leave and go away they almost invariably die. There have been several instances in the East where the system has been accustomed to the poison, and the reaction from leaving this atmosphere is so great that death has been the result.

Dr. Chapman—This shows what a miserable condition our sewers are in.

Dr. McNeil—This is also an explanation that will meet the case. There is one point, however, that may be brought out here. The bacteriologists of Paris say that the sewers are free from bacteria.

Dr. Martin—That is so in Paris, but it is not so here.

Dr. Chapman—One more question for Dr. McNeil. What is the antidote for Quinine?

Dr. McNeil—There is no one antidote. In choosing precisely an antidote for disease there is only one guide to follow, and that is the law of similars. In the *Materia Medica Pura* we find that there are twenty-three drugs to antidote the effects of Mercury, and they are not given indiscriminately. For salivation there is one kind of drugs; for another condition another set of drugs, and so on, following the law of similars. *Arsenicum* is a deep-acting drug, and is often of use, though *Natrum-mur.* more frequently. *Pulsatilla* and *Rhus-tox.* in milder cases. Suppose a patient had been taking large doses of Quinine, and he wanted treatment without it, and you knew that he had been taking these large doses. If the symptoms called for it I would give Sulphate of Quinine.

Dr. Chapman—If the patient should not exhibit symptoms calling for Sulphate of Quinine?

Dr. McNeil—If he did I would give it. This is a knotty question. Of one point I am sure: a drug does not always produce the same effects. I was poisoned with Rhus-tox. I took *Rhus* high as well as several other remedies, but none of them did any good. I worked it out with Bœnninghausen, and found that *Sulphur* was the remedy, which cured in a short time. In cases of Quinine cachexia *Nutrum-mur.* or *Arsenicum* have frequently cured. As regards this idea of Bœnninghausen and Sawyer using high potencies as antidotes, I say that a high potency is an antidote, but not necessarily *the* antidote. There may be others.

Dr. Chapman—The high potency of the same drug must be the antidote. It is impossible, for instance, for Mercury to produce Sulphur symptoms.

Dr. Martin—There is one condition to be considered, and that is the individual idiosyncrasy. For instance, Rhus produces a certain set of symptoms in certain individuals. I had three cases of poison-oak, and the symptoms were all different. The patients were of one family, and yet *Sulphur*, *Graphites*, and *Rhus* were indicated. They are all getting well. In thinking it out, I took into account the individual idiosyncrasies of these patients.

Dr. McNeil—Thank you. This completes my chain.

Dr. Chapman—The antidote of the poison is the drug itself. This seems plausible to me. I cannot understand the other idea.

Dr. Lilienthal—In proving a drug upon half a dozen people, do you get the same organs affected in each one? Some organs may be affected in one, and some in another.

Dr. Chapman—When the patient is susceptible you will get the characteristic symptoms of the drug.

Dr. Lilienthal—What are the characteristic symptoms?

Dr. Chapman—That depends upon the drug given. If *Rhus* you would find symptoms better from motion, worse at night, etc.

Dr. Lilienthal—Those are concomitant symptoms.

Dr. Chapman—They are the leading symptoms of that drug.

Dr. McNeil—Dr. Chapman is going into philosophy. When theories get in the way of facts, the theories are liable to be damaged. *Rhus* failed in my case; *Sulphur* cured. When I

worked it out, *Sulphur* was 17, *Phosphorus* 15, and *Calcarea* 25. *Rhus* was away down.

Dr. Chapman—In such a case you suffered more from psora than from *Rhus*.

Dr. McNeil—That is an objection likely to arise. It was the first time that *Sulphur* ever did me any good. An illustration: Smith and Jones poisoned by Rhus. There will be two forces in both cases, namely, Smith and Rhus in one case, and Jones and Rhus in the other case. The result in both cases will be different. The individuality of Jones and Smith will present itself like the cases of Dr. Martin, which required *Rhus* for one case, *Sulphur* for another, and *Graphites* for another.

Dr. Chapman—A psoric case is not a fit case to prove drugs upon. They simply arouse the dyscrasia or miasm. It is impossible to get a reliable drug proving in these cases.

Dr. Lilienthal—If that were so, and to a great extent it is so, we would have to shut down upon our present provings, as we could not find any one pure enough to prove upon. We get several or more to prove upon, and from all get a pure proving of the drug.

Dr. Chapman—I said those that are markedly tainted.

Dr. McNeil—Hahnemann agrees with you on that.

Section 239 was then read.

### Discussion.

Dr. McNeill—It has been frequently stated that there have been but few drugs proven in intermittents. Bœnninghausen gives one hundred and fifty different drugs. A patient of mine in Michigan, a cultured Eastern lady, came to me in the fall of the year. I gave fifteen grains of Quinine without any perceptible effect. Fortunately for me and for her, cold weather came on and she felt better. Early in the spring she was almost frantic with a most violent paroxysm of intermittent fever. During the winter she had had eczema, and I had given her *Graphites* in moderate potencies. Before the attack of intermittent fever came on I had sent for *Graphites*⁴ and had the most satisfactory results. No one would think of *Graphites* for intermittent fever. I have not had a case since with indications for *Graphites.* Old-school practitioners are fortunate in this disease as well as in other diseases. In wiry patients they give Sulphate of Quinine in ten, fifteen, and one hundred grains doses in all cases. We question our patients by the *Repertory* or out of our heads, and then give a remedy, and it is much harder work than that of our opponents, the allopaths.

Section 240 was then read.

DISCUSSION.

Dr. McNeil—This proves that in acute diseases, if the remedy is not given promptly, the latent psora will be aroused, and an anti-psoric remedy will be necessary.

Section 241 was next read.

DISCUSSION.

Dr. McNeil—My opinion comes in here that this is the corner-stone of success. Similar causes must have like effects, except as modified by the individual characteristics. If we breathe the same air there will be similar effects, being produced by the same cause. In the beginning of the disease, before the psora is aroused, the individuality of the person excepted, they will have the same line of symptoms. The paroxysms may be quotidian, every day or every other day, but there will be something in common between them. Malaria is not a unit. It is not the same to-day, yesterday, and forever. There is a difference at different seasons, etc., hence different remedies are necessary. In a case of intermittent fever with, perhaps, only one paroxysm, when this first paroxysm does not give a clear picture of the disease, but you know the epidemic character of it, you may give the indicated remedy with good effect. Patients get well themselves, we say, but this is not so. They may get well in ten or eleven years.

Dr. Chapman—I have seen homœopathic remedies cure ague many times. I want to hear from Dr. McNeil on congestive chills.

Dr. Lilienthal—I wish to ask Dr. Selfridge if he has ever examined the blood of persons suffering from malaria? It is claimed that one can see the gradual shrinking and disappearance of the organisms of malarial poisoning, when the patient is under the regular treatment. It would be interesting to know what effect homœopathic treatment would have upon these organisms.

Dr. Chapman—I believe that our homœopathic remedies put the blood in such a condition that these germs cannot live there.

Sections 242 and 243 were then read.

### Discussion.

Dr. McNeil—In regard to congestive chills, in the Valley of the Ohio it was a common thing to hear that "Smith died of congestive chill." From my experience these cases should be called "Quinine intermittents." I have known of only two cases of congestive chill occurring in persons who had not taken Quinine. One case, a Mrs. Kelly, who had a congestive chill, with aggravation from moving, the vomiting was not only aggravated by moving, but also by sitting up. I gave her Bryonia. I myself had a congestive chill. I went to bed alone; was extremely restless; felt that the bed was not big enough for me; vomited; most terrible anguish; couldn't stay in bed; great thirst, with aggravation from drinking. Toward morning took Arsenicum$^{30}$ or the 200th. It was not below the 30th. I was weighed the day before. The next day I could not go to the office. The second day I was weighed again, and was twelve pounds lighter than I was the day before I was taken sick. This was certainly a congestive chill. One case of intermittent, the patient was given forty-five grains of Quinine on Thursday, and was dead Sunday with congestive chills.

Dr. Chapman—Do I understand that congestive chills come only from the use of Quinine.

Dr. McNeil—This Mrs. Kelly and myself are the only ones that I know of who had not taken Quinine.

Dr. Martin—Why do you call yours a case of congestive chill?

Dr. McNeil—That is what they called it in the Ohio Valley.

Dr. Martin—In a real congestive chill there are symptoms pointing to congestion of internal organs; there is usually delirium or unconsciousness. It is apt to be of nervous origin, and there is a contraction of the superficial blood-vessels which produces the coldness and the internal congestion. These cases are nearly always fatal. Often, lay people and physicians call a severe chill a congestive chill, but it is not correct. It is a loose way into which physicians have fallen in regard to the use of this term.

Dr. J. M. Selfridge—The term is a misnomer. Any chill is congestive.

Dr. McNeil—This is simply the name given to these conditions in the Ohio and Mississippi Valley. It is not necessarily a point for discussion just here.

Dr. Martin—True congestive chill is different from that described by Dr. McNeil as existing in the Ohio Valley.

Dr. Chapman—Drs. Allen and Fisher had a discussion in the *Advance* in regard to this matter. Dr. Fisher said that the cases were unmanageable, and that you must give Quinine.

Dr. McNeil—Give us some of your own experience, Dr. Chapman.

Dr. Chapman—I have never seen a clear case of congestive chill; I mean malarial.

Dr. J. M. Selfridge—Wood calls it "pernicious fever."

Dr. Martin—A congestive chill is not always due to malaria. I have seen three cases in my experience, and none of them had ever had m ılaria. They died one-half hour after the chill commenced.

Dr. McNeil—You mean congestive chill of nervous origin.

Dr. Chapman—We are simply discussing malaria.

Dr. J. M. Selfridge—An old idea in the Ohio Valley is that you will live through the first chill, and may live through the second, but will surely die in the third.

Section 244 was next read.

### Discussion.

Dr. Chapman—That is the nearest to the routine treatment that I have ever noticed in Hahnemann's writings.

Dr. McNeil—I am not surprised that Dr. Chapman should say this. Hahnemann is wrong. In one respect Hahnemann's life was too short. There was too much to be done to complete all that he set out to do. Bœnninghausen corrected many mistakes that he made. Bœnninghausen gives one hundred and fifty drugs for intermittents. *Cinchona* is not for all cases, as is shown by Bœnninghausen.

Dr. Chapman—It seems strange to me that he should here make such an assertion.

Dr. McNeil—This is a contradiction of the rest of his works. We must follow the rule of interpretation, and consider what goes before and what follows a certain statement. We can tell by the general trend of his works what he wishes to convey.

Dr. Martin—Here it says that *Cinchona* may not relieve, but that the anti-psorics will.

Dr. McNeil—The reason for this is because it is not a specific. The remedy may be *Bryonia*, *Arsenicum*, *Natrum-mur.*, *Ipecac.*, etc. All marsh intermittents are not alike.

Dr. J. M. Selfridge—He would not give *Cinchona* if he thought another remedy was indicated.

Dr. McNeil—*Bryonia*, *Rhus*, *Arsenicum*, or *Natrum-mur.* may do what he says *Cinchona* will.

Dr. J. M. Selfridge—The point you make is that he pins his faith to one remedy or to an anti-psoric. A little further back he gives a list of other remedies.

Dr. Underwood read a clause in Section 243.

Dr. Chapman—He says that certain conditions are of psoric origin and require anti-psoric remedies.

Dr. McNeil—There are certainly cases of intermittent fever that are cured by non-anti-psoric remedies.

Dr. J. M. Selfridge—Are they then of anti-psoric origin?

Dr. McNeil—No. I think if we read *The Organon* carefully in the original we would not be apt to run into these snarls. The latent psora becomes aroused, and then these conditions require anti-psoric remedies.

Dr. Chapman—In all manifestations of disease of psoric origin are anti-psoric remedies required?

Dr. McNeil—Yes; in order to get a cure. An illustration: Say a rheumatic case where *Rhus* is indicated. If given early it would do wonders. In a month, more or less, with the same symptoms, if *Rhus* is given high, it may do good, but often not. *Natrum-mur.* might then complete the cure.

Dr. C. M. Selfridge—In a rheumatic case *Rhus* helped for awhile, and then *Psorinum* removed the psora that came out, and then *Rhus* cured.

Dr. Martin—In *The Organon* Hahnemann says that all diseases are classed under three heads: syphilis, sycosis, and psora; and the greateat of all is psora.

Dr. McNeil—Hahnemann does not mean that they are all classified under these three heads; because, if they were, we would have no use for *Rhus*, etc. In cases that have run on for some time the psora is aroused, and then anti-psoric remedies are indicated.

Dr. Martin—Is giving an anti-psoric remedy for a psoric disease true Homeopathy?

Dr. McNeil—I will answer that question at our next meeting.

On motion of Dr. McNeil, seconded by Dr. Wilson, the meeting then adjourned to meet at the office of Dr. J. E. Lilienthal, in San Francisco, the third Friday in August, when the reading of *The Organon* would be commenced at Section 245.

Reported by ELEANOR F. MARTIN, M. D.

